# Annotations

**Published:** 1910-04-09

**Authors:** 


### Annotations.

### The late Dr. W. B. Cheadle.

By the death of Dr. Cheadle, announced in our last week's issue, not only does London lose a very eminent physician but the nation loses one who in his own unostentatious way has contributed to the development and prosperity of our greatest colony. To the present generation Dr. Cheadle was best known as consulting physician to St. Mary's Hospital and to the Great Ormond Street Hospital for Sick Children. In both these rôles his personality secured for him the respect and affection of those who worked under him, in whatever capacity. But nearly fifty years ago it was as a pioneer and traveller in the then unexplored Canadian North-West that the young physician attracted attention. In 1862 the Viscount Milton of those days, a young man of delicate health but ambitious spirit, set out from England with the subject of this notice as his companion, ostensibly for a buffalo-shooting trip to the prairies of the Saskatchewan. In reality he had, on a previous expedition, conceived the idea of pushing on into the unknown, crossing the Rocky Mountains, and ending up by traversing British Columbia to Vancouver; and this plan he and Dr. Cheadle proceeded, with very inadequate equipment, to put into execution. After narrowly escaping massacre at the hands of the then unconquered Red man, and after shooting many buffalo for food, they crossed the mountains fairly easily. At the far side, however, they had to cut their way through virgin forests, and therein the whole party would probably have died but for the courage and physical strength of Dr. Cheadle. In the account of their adventures which was subsequently published it is recorded that the latter, on at last reaching a land of plenty, gained three stone in weight in a fortnight. It was in this book that the feasibility and the advantages of a trans-continental railway were first pointed out; and the Canadian Pacific Railway, with the enormous development of all the North-West, which has followed, was the direct outcome of this journey. Since no public recognition of his service to civilisation in this respect has ever been accorded to the deceased physician, it is the more necessary that the medical profession should not let his memory altogether die in the land.

### Race and Infant Mortality.

Some curious conclusions have been published in the *Boston Medical and Surgical Journal* by Drs. Cabot and Ritchie as the result of a research in that city into infant mortality among the various races which contribute to its population. Contrary to common experience in London, it seems that the Jews far exceed all others in the number of stillbirths and in respect of diseases of the digestive system fatal during the first two years of life. The Italian babies suffer but little from birth accidents, prematurity and congenital weakness, and gastro-intestinal diseases; but greatly from general diseases, amongst which pneumonia stands easily first. The Irish have a better record than either the Jews or the Italians in respect of all these causes of mortality, and in their immunity from prematurity they surpass also the native-born "Americans." The latter, however, have the advantage over all the immigrant races in every one of the causes of infant mortality except prematurity, in which they stand next to the Jews, and lose far fewer infants per thousand than any other race. This may be explained by their longer settlement in the country, or, more probably, by a restriction of procreation which enables them to give greater attention to those infants who are born. The facts are instructive, and not all quite easy of explanation.

### Hospital Contracts.

Under this heading the first article of a new series appears in our columns to-day. It is hardly too much to say that up till the present time no information has been easily accessible for the managers of hospitals and other institutions in regard to one of the most important duties they have to perform—purchase of the best goods in the cheapest market. It will be our aim to let a little light into the cloudy region of contracts, and to pave the way for a complete understanding of the gentle art of getting the best for the money. Buying is entirely expert work, and it is therefore not surprising that big blunders are made every day by people skilled in a hundred different directions, but untrained to distinguish good from bad, or dear from cheap, in the common objects of the breakfast table, the linen-room, or the dispensary. Glancing through the suggestions furnished in regard to provisions alone, everyone will appreciate the fact that this is a wider matter than is commonly supposed. There are pitfalls in regard to inviting tenders, and more in the manner of accepting them. There are difficulties of storage, difficulties in keeping good supplies up to the mark, difficulties in rejecting bad supplies. There is the danger of getting things too dear; there is danger even in getting them too cheap. The managers are, in fact, dealing with smart commercial men, keenly stimulated by competition to take all the advantage they can get, and often blunted in their sense of what constitutes fair dealing. With all this, the prices are constantly changing, as well as the sources of supply. New countries compete for our custom, and accepted traditions as to the best brands to purchase may have become empty prejudices by the expiration of the year. Some of our readers have a wide and thorough knowledge of the markets for institution purposes, and others are seeking information. Through the medium of this series we shall hope to enable them to reach one another, and the resulting discussion cannot fail to be of interest to all. The latest information on markets and prices will be furnished by experts, and the whole subject of contracts will be treated in an exhaustive manner adapted to the special requirements of both large and small institutions.

